# Regulation of *Pseudomonas aeruginosa* Virulence by Distinct Iron Sources

**DOI:** 10.3390/genes7120126

**Published:** 2016-12-14

**Authors:** Alexandria A. Reinhart, Amanda G. Oglesby-Sherrouse

**Affiliations:** 1 Wound Infections Department, Bacterial Diseases Branch, Walter Reed Army Institute of Research, Silver Spring, MD 20910, USA; alexandria.a.reinhart.ctr@mail.mil; 2 Department of Pharmaceutical Sciences, School of Pharmacy, and Department of Microbiology and Immunology, School of Medicine, University of Maryland, Baltimore, MD 21201, USA

**Keywords:** *Pseudomonas aeruginosa*, heme, siderophores, iron, small RNAs, biofilms

## Abstract

*Pseudomonas aeruginosa* is a ubiquitous environmental bacterium and versatile opportunistic pathogen. Like most other organisms, *P. aeruginosa* requires iron for survival, yet iron rapidly reacts with oxygen and water to form stable ferric (FeIII) oxides and hydroxides, limiting its availability to living organisms. During infection, iron is also sequestered by the host innate immune system, further limiting its availability. *P. aeruginosa’s* capacity to cause disease in diverse host environments is due to its ability to scavenge iron from a variety of host iron sources. Work over the past two decades has further shown that different iron sources can affect the expression of distinct virulence traits. This review discusses how the individual components of *P. aeruginosa’s* iron regulatory network allow this opportunist to adapt to a multitude of host environments during infection.

## 1. Introduction

*Pseudomonas aeruginosa* is a ubiquitous soil bacterium and versatile opportunistic pathogen, capable of causing life-threatening acute and chronic infections in a variety of patient populations. This includes acute lung and blood infections in cancer patients [1,2,3] and 10% of all nosocomial infections [4]. *P. aeruginosa* also causes life-long chronic lung infections in individuals with cystic fibrosis (CF) [5] and is a significant contributor to chronic wound infections in diabetics and surgical patients [6,7]. *P. aeruginosa* employs a large armament of virulence factors to survive in these different host environments and cause disease, and the impact of individual virulence traits varies by infection type (reviewed by [8,9,10]). For example, the *P. aeruginosa* type three secretion system is a critical virulence factor during acute lung infections [11,12], but is downregulated in chronic *P. aeruginosa* isolates from the lungs of CF patients [13]. In contrast, overproduction of alginate, a polysaccharide that allows for mucoid biofilm formation, occurs almost exclusively during chronic CF lung infections [14]. These adaptations are dependent upon regulatory pathways that coordinate the expression of diverse virulence traits in response to specific host stimuli, demonstrating the critical role of gene regulation in *P. aeruginosa* pathogenesis.

Iron is an abundant and essential metallonutrient for almost all organisms. Despite its abundance in the Earth’s crust, iron rapidly reacts with oxygen and water to form stable ferric (Fe(III)) oxides and hydroxides (e.g., rust), limiting its availability to living organisms. Pathogenic bacteria face an additional barrier to iron acquisition during infection due to sequestration by host innate immune factors, a process referred to as “nutritional immunity” [15,16]. *P. aeruginosa* can overcome this host immune response to acquire iron through several mechanisms (reviewed in [17]), many of which have established roles in pathogenesis [18,19,20,21,22,23]. Due to the exceedingly low concentration of iron in the mammalian host, iron depletion also serves as a robust signal for *P. aeruginosa* to express virulence-related genes (reviewed in [24,25]). Iron regulation in *P. aeruginosa* occurs through a complex regulatory network capable of incorporating numerous environmental stimuli, ensuring timely expression of individual iron uptake systems and virulence factors. The ferric uptake regulatory (Fur) protein, which has been extensively reviewed elsewhere [24,26,27,28], presides over much of this regulatory hierarchy and controls multiple aspects of *P. aeruginosa* virulence. Since the last comprehensive reviews on this topic [17,24,28,29], several new discoveries have been made regarding how individual iron regulatory pathways affect different aspects of *P. aeruginosa* physiology and virulence. This review discusses some of these more recent findings and places them in the context of the larger body of work on how iron regulates *P. aeruginosa* pathogenesis.

## 2. Iron and Host–Pathogen Interactions

Successful infection by most microbial pathogens is dependent upon their ability to scavenge iron from the host, which sequesters this nutrient away from invading pathogens. *P. aeruginosa* is adept at overcoming this host response, possessing multiple systems that can scavenge host iron. The majority of iron in the human body is contained by hemoglobin in the form of heme [30], which can be acquired by *P. aeruginosa* through two distinct outer membrane proteins, PhuR and HasR, and degraded by a cytoplasmic heme oxygenase, HemO [31]. Ferric iron (Fe(III)), which is predominant in aerobic environments and rapidly reacts to form insoluble ferric hydroxides, is sequestered by host proteins transferrin and lactoferrin in the blood and at mucosal surfaces, respectively [32]. *P. aeruginosa* synthesizes and secretes two distinct siderophores, pyoverdine and pyochelin, which have been extensively reviewed elsewhere [9,33]. Each of these siderophores is capable of scavenging ferric iron from host proteins and is thus required in acute infection models [18,19,20,21]. In hypoxic environments, such as those found in the CF lung and in biofilms, iron is mainly found in its reduced ferrous form (Fe(II)), which can be acquired by *P. aeruginosa* through the Feo system [34,35]. The multiplicity of iron uptake systems is likely reflective of the multiple environments that *P. aeruginosa* can inhabit, underlying its versatility as an opportunistic pathogen. As outlined below, each of these sources of iron can initiate distinct regulatory pathways that also affect virulence, interlinking iron uptake and virulence gene expression in diverse infection states.

## 3. Coordinated Regulation of Virulence and Iron Uptake

### 3.1. Pyoverdine-Mediated Regulation of Virulence

**Figure 1:**
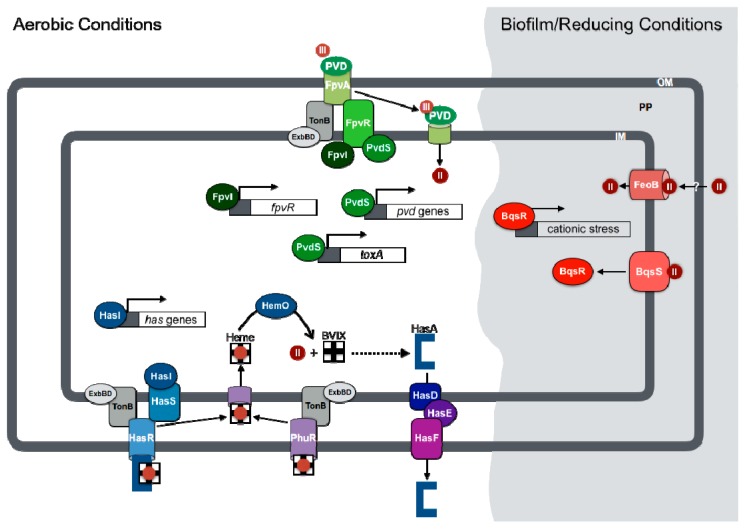
Overview of iron regulation of virulence in *Pseudomonas aeruginosa*. Iron regulation in *P. aeruginosa* is largely mediated by ferric uptake regulatory protein (Fur)-responsive signal transduction systems that respond to specific sources of iron. This includes the sigma factors that regulate pyoverdine biosynthesis and secreted toxins (PvdS), pyoverdine uptake (FpvI), and heme acquisition (HasI). Heme degradation by the HemO heme oxygenase may also contribute to gene regulation through the production of the β or δ biliverdin (BVIX) metabolites. In anaerobic and reducing environments, such as those found in biofilms, ferrous iron (Fe(II)) can initiate a distinct set of regulatory pathways through the BqsSR two-component regulatory system. Known regulatory pathways are shown by a solid line, and putative pathways are shown by a dashed line. OM: outer membrane; PP: periplasm; IM: inner membrane. II and III represent ferrous and ferric iron, respectively.

The expression of genes encoding iron uptake systems in *P. aeruginosa* is presided over by the Fur protein [36,37,38]. In iron-replete environments, Fur binds to iron and becomes an active transcriptional repressor, blocking the expression of genes for iron acquisition, as well as multiple virulence traits [39,40,41]. Some of the earliest evidence that Fur could regulate the expression of virulence factors by *P. aeruginosa* resulted from studies of exotoxin A [42]. Exotoxin A is a potent A–B exotoxin that mediates its entry into target host cells through its cell-binding (B) domain, then ADP ribosylates host elongation factor 2 (EF-2) to block protein synthesis through its enzymatic (A) domain [43]. Expression of exotoxin A is indirectly regulated by the Fur protein [40,44] through a pyoverdine-specific cell surfacing signaling (CSS) system [45]. In this CSS system, the FpvA outer membrane receptor transports ferric-pyoverdine into the periplasm, harnessing energy from the inner membrane through the TonB-ExbBD protein complex (Figure 1) [46]. Binding of pyoverdine to FpvA simultaneously initiates a signaling cascade through the FpvR anti-sigma factor. FpvR controls the activity of two extracytoplasmic function (ECF) alternative sigma factors, PvdS and FpvI. The FpvI sigma factor recruits RNA polymerase (RNAP) to the promoter of the gene for the FpvA outer membrane receptor, while PvdS promotes the expression of genes for pyoverdine biosynthesis and exotoxin A (*toxA*) (Figure 1) [47]. Fur directly represses the *pvdS* gene, resulting in downregulation of the entire pyoverdine uptake and regulatory system in iron-replete conditions.

Currently, it remains unclear if PvdS affects the expression of *toxA* by directly binding to its promoter. However, PvdS is known to directly bind to the gene promoters of two upstream regulatory proteins that promote the expression of *toxA*: RegA and PtxR [45,48,49]. PvdS also promotes the expression of two secreted proteases, an endoprotease (PrpL) [39] and an alkaline protease (AP) [50], which may contribute to infection by promoting tissue invasion, modulating immune signaling, and enhancing nutrient acquisition. Thus, pyoverdine functions not only as a ferric iron-scavenging molecule, but as a signaling molecule that can promote the expression of virulence factors in response to the successful scavenging of ferric iron. A recent report demonstrated that this signaling function is critical for virulence by mutating the gene for the FpvR anti-sigma factor in a pyoverdine biosynthetic knockout mutant background (∆*pvdA*). While the ∆*fpvR*∆*pvdA* mutant cannot produce pyoverdine, it shows the restored production of virulence factors due to loss of the FpvR anti-sigma factor, allowing constitutive PvdS activity. As a result, the ∆*fpvR*∆*pvdA* double mutant is significantly more virulent than the ∆*pvdA* single mutant in an airway infection model [51]. Thus, signaling cells to induce the expression of virulence factors appears to be a critical function of the pyoverdine siderophore during infection.

### 3.2. Ferrous Iron Regulation of Biofilm Formation and Antibiotic Resistance

Biofilm formation is a typical mode of growth during chronic *P. aeruginosa* infections, which are characterized by hypoxic and reducing conditions that result in the reduction of iron to its more bioavailable ferrous state [34]. Ferrous iron acquisition is mediated in part by *feoB*, encoding a putative G protein-like inner membrane permease, and potentially other inner membrane transporters [35,51]. The *feoB* gene was additionally shown to facilitate biofilm formation in the presence of phenazines, small metabolites produced by *P. aeruginosa* that can reduce ferric iron to its ferrous oxidation state [52]. The *feoB* gene is repressed by iron, and its promoter contains a consensus Fur binding site, indicating that its expression is directly regulated by the Fur protein [53]. Intriguingly, ferrous iron initiates a regulatory cascade that is distinct from the Fur regulon through the BqsSR two-component regulatory system [54], which was originally identified as a positive regulator of biofilm dispersal [55]. A recent report from Newman and colleagues further demonstrated the impact of this system on mediating resistance to cationic stress in response to ferrous iron [56]. Specifically, BqsSR was shown to promote the expression of numerous genes involved in polyamine synthesis and transport. They further showed that supplementation of cultures with one specific type of polyamine, spermidine, could protect a ∆*bqsSR* mutant from cationic stresses. The implications for this ferrous iron regulatory pathway in virulence are particularly notable when considering the cationic properties of many antimicrobial compounds used to treat chronic *P. aeruginosa* infections, such as polymixins and tobramycin [57].

### 3.3. Heme-Dependent Regulation of Gene Expression

Heme is the most abundant form of iron in the human body [30]. Thus, its potential to serve as both an iron source and a signaling molecule during infection are of great interest. Moreover, numerous studies suggest that heme may become an increasingly relevant source of iron during CF lung infections [58,59,60]. *P. aeruginosa* possesses two distinct outer membrane transporters for heme, PhuR and HasR, which mediate distinct roles in heme uptake. Metabolic studies recently demonstrated that PhuR is the major heme transporter of *P. aeruginosa*, while HasR functions primarily as a heme receptor with signaling capacities [61]. HasR is thought to transport heme captured by the HasA-secreted hemophore protein, as a part of a distinct CSS system that was originally described in another Gram-negative pathogen, *Serratia marcescens* [62]. In *S. marcescens*, binding of the heme-loaded HasA hemophore to the HasR outer membrane receptor initiates a signaling cascade through the HasS anti-sigma factor and HasI ECF sigma factor [63]. The genes for the Has system are conserved in *P. aeruginosa*, and therefore HasR is hypothesized to initiate a signaling pathway that auto-regulates the expression of genes for heme acquisition, and potentially virulence, in *P. aeruginosa*.

In addition to the HasR CSS system, heme may also regulate gene expression through metabolic feedback. Once internalized, heme is degraded by the iron-regulated HemO heme oxygenase to biliverdin, releasing carbon monoxide and iron [64,65]. Notably, the biliverdin isomer pattern produced by *P. aeruginosa* HemO is unique in that the heme molecule is cleaved at the β or δ carbon of the porphyrin ring, in contrast to cleavage at the α carbon mediated by all other characterized heme oxygenases [66]. The unique biliverdin isomer pattern produced by *P. aeruginosa* HemO was recently shown to allow for the positive feedback regulation of HasA hemophore production [67]. Combined, these studies indicate that the heme acquisition pathway of *P. aeruginosa* is capable of regulating gene expression by at least two distinct mechanisms. While the impacts of these systems on virulence gene expression are not yet known, earlier studies showed that inhibition of HemO attenuated virulence of *P. aeruginosa* in a *Caenorhabditis elegans* model of infection [68]. More studies are clearly needed to understand the full impact of heme-mediated gene regulation during *P. aeruginosa* pathogenesis.

## 4. Post-Transcriptional Regulation by Iron-Responsive Small RNAs

In addition to the negative regulation of genes for iron uptake systems and virulence, the *P. aeruginosa* Fur protein mediates indirect positive regulation through the negative regulation of two non-coding small RNAs (sRNAs) named PrrF1 and PrrF2 [69]. The PrrF sRNAs are orthologous to RyhB, the first iron-regulated sRNA to be discovered, in *Escherichia coli* [70]. Both the PrrF and RyhB sRNAs repress the expression of a large repertoire of non-essential iron-containing proteins produced by their respective organisms, including the SodB superoxide dismutase and several tricarboxylic acid cycle enzymes containing iron-sulfur clusters. Regulation by the PrrF sRNAs occurs post-transcriptionally [69] and is thought to proceed in a manner similar to that of RyhB, which binds to complementary regions of mRNA molecules to reduce their stability and translation [71,72]. Negative regulation of iron-containing proteins by RyhB was previously shown to promote growth in iron-depleted conditions by increasing intracellular pools of iron, an activity dubbed the “iron sparing response” [73]. The PrrF sRNAs mediate a similar response in *P. aeruginosa* and were recently shown to be required for virulence, highlighting a novel role for iron regulation in *P. aeruginosa* pathogenesis [74].

While the maintenance of iron homeostasis is likely critical for virulence, it remains unclear if the requirement for the PrrF sRNAs during infection is solely due to the iron-sparing response it mediates. The PrrF sRNAs were previously shown to be required for the optimal production of the *Pseudomonas* quinolone signal (PQS) [75], a quorum-sensing molecule that activates the expression of several virulence genes [76,77]. This effect is achieved through repression of the *antABC* and *catBCA* mRNAs, encoding enzymes that metabolize anthranilate, which also serves as the biosynthetic precursor for PQS [78]. PrrF regulation of anthranilate degradation is thought to occur through the direct repression of *antR*, encoding a LysR-type transcriptional regulator that activates the transcription of *antABC* and *catBCA* in the presence of anthranilate [75]. Thus, the impact of PrrF regulation on cell physiology extends beyond iron homeostasis, potentially exerting effects on virulence gene expression.

**Figure 2:**
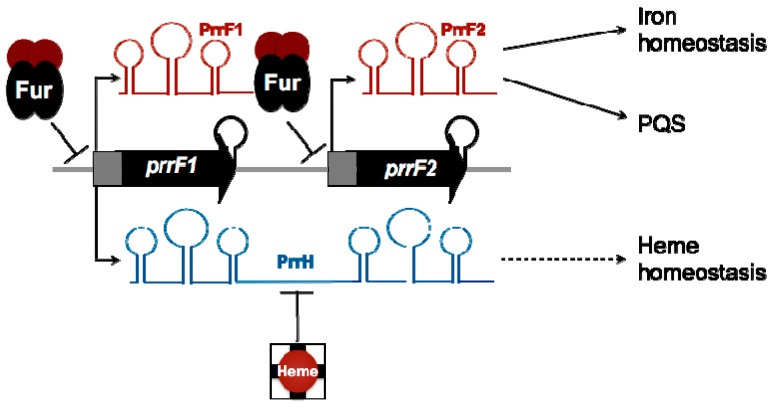
Regulation by the PrrF and PrrH small RNAs (sRNAs). The PrrF sRNAs are transcribed from two highly homologous genes on the *P. aeruginosa* chromosome. PrrF contributes to both iron homeostasis and production of the PQS quorum-sensing molecule through negative regulation of several iron-containing proteins. The tandem duplication of *prrF*1 and *prrF*2 genes allows for the production of a distinct sRNA, PrrH, which is responsive to heme. PrrH is hypothesized to mediate heme homeostasis by regulating a distinct set of genes.

The tandem organization of the *prrF* genes on the *P. aeruginosa* chromosome allows for the expression of a third, heme-responsive sRNA named PrrH (Figure 2), suggesting yet another mechanism by which the *prrF* locus, and heme, can contribute to the regulation of virulence [79]. PrrH is hypothesized to regulate a specific subset of genes involved in heme metabolism (i.e., synthesis and degradation), heme uptake (acquisition of extracellular heme), and other cellular processes, via its unique sequence derived from the *prrF1–prrF2* intergenic region (Figure 2). One potential target of PrrH is *nirL*, encoding a regulator of genes for dissimilatory nitrite reductase (NIR) and its heme d_1_ prosthetic group [79]. Biosynthesis of heme d_1_ branches from the central heme biosynthetic pathway with NirF, NirJ, and NirE catalyzing its production from uroporphyrinogen III [80]. Thus, repression of NIR biosynthesis by PrrH may prioritize the function of the heme biosynthetic pathways under limiting heme concentrations, contributing to heme homeostasis [80]. Additional putative targets of the PrrH sRNA include *vreR*, which encodes a receptor for a CSS involved in virulence gene regulation, and *phuS*, which mediates trafficking of transported heme to HemO [81,82,83]. However, the role of PrrH and its regulation of these putative gene targets in pathogenesis remain unknown.

## 5. Iron Regulation of Biofilm Formation

Multiple studies have shown that iron supplementation has a positive impact on *P. aeruginosa* biofilm formation [84,85,86], and that this regulatory effect is dependent upon the Fur protein [85]. While the precise mechanism of iron-regulated biofilm formation remains unclear, several studies have shown that iron can affect the expression of multiple factors that affect biofilm formation. For example, iron negatively affects the production and secretion of rhamnolipid, a type of biosurfactant that promotes swarming motility and prevents the formation of mature biofilms [87]. Iron also promotes the expression of genes for the synthesis of the Psl exopolysaccharide, a major component of *P. aeruginosa* biofilms [88]. In contrast, iron negatively impacts the production of alginate, a distinct exopolysaccharide important for mucoid biofilms that form in the lungs of CF patients [89]. Thus, iron and Fur likely play a complex role in modulating biofilm production during chronic lung infections. The bioavailability of iron within the CF lung also changes dramatically over time due to chronic infection and inflammation [34,58,59,90]. Mutations in the CF transmembrane receptor (CFTR), representing the underlying cause of CF disease, result in further release of iron into the extracellular space, thus increasing biofilm formation by *P. aeruginosa* [91]. Considering that biofilm formation protects *P. aeruginosa* from antimicrobial treatment, there may be therapeutic potential in blocking iron uptake during chronic infection to enhance the efficacy of current antibiotics [86,92,93,94,95].

## 6. Future Outlooks

Iron acquisition has long been appreciated as a critical mediator of *P. aeruginosa* virulence, and the studies reviewed here demonstrate how iron regulatory pathways also have the capacity to affect *P. aeruginosa* pathogenesis. In particular, these studies outline the role that different iron sources play in modulating the expression of diverse virulence traits, such as exotoxin production, antibiotic resistance, and biofilm formation, underlying the amazing versatility of this pathogen. Because of the broad impacts of this regulatory network on virulence, it is also important to consider the role that iron regulation may play in the development of novel therapeutic strategies that target iron uptake. For example, iron chelation has been proposed as a potential therapy in CF patients to block biofilm formation, due in part to the availability of Food and Drug Administration-approved ferric iron chelators such as desferasirox and desferrioxamine [92]. However, such an approach could potentially enhance the production of other virulence factors, resulting in increased host damage. In contrast, iron chelation approaches that target ferrous iron, which is more predominant in the CF lung, may prove safer and more efficacious than currently approved ferric iron chelators.

Alternatively, it may be possible to modulate the iron regulatory networks of *P. aeruginosa* to block virulence. One promising approach toward this goal is to knock down the expression of the PrrF sRNAs, which are required for virulence, using antisense oligonucleotides (AS-ODNs) [96,97,98]. Bacterial sRNAs are important regulators of virulence in many bacterial species [74,99,100], and the iron-regulated sRNAs of other bacterial species, including *Klebsiella pneumoniae* [101], uropathogenic *E. coli* [102], and *Shigella* species [103,104], have been shown to modulate virulence traits. Thus, this approach could potentially be modified to treat a large number of infectious agents, exerting a broad impact on the clinical management of other antibiotic-resistant organisms.
